# High‐Field NMR, Reactivity, and DFT Modeling Reveal the γ‐Al_2_O_3_ Surface Hydroxyl Network**

**DOI:** 10.1002/anie.202207316

**Published:** 2022-08-03

**Authors:** Nicolas Merle, Tarnuma Tabassum, Susannah L. Scott, Alessandro Motta, Kai Szeto, Mostafa Taoufik, Régis Michaël Gauvin, Laurent Delevoye

**Affiliations:** ^1^ Univ. Lille, CNRS Centrale Lille Univ. Artois, UMR 8181, UCCS, Unité de Catalyse et Chimie du Solide 59000 Lille France; ^2^ Department of Chemistry & Biochemistry and Department of Chemical Engineering University of California, Santa Barbara Santa Barbara CA 93106 USA; ^3^ Dipartimento di Scienze Chimiche Università di Roma “La Sapienza” and INSTM, UdR Roma Piazzale Aldo Moro 5 00185 Roma Italy; ^4^ Univ. Lyon 1, CPE Lyon, CNRS UMR 5265 Laboratoire de Chimie Catalyse Polymères et Procédés (C2P2) Université de Lyon 69616 Villeurbanne France; ^5^ Chimie ParisTech PSL University, CNRS Institut de Recherche de Chimie Paris 75005 Paris France

**Keywords:** Alumina, Catalysis, Density Functional Calculations, Solid-State NMR, Surface Chemistry

## Abstract

Aluminas are strategic materials used in many major industrial processes, either as catalyst supports or as catalysts in their own right. The transition alumina γ‐Al_2_O_3_ is a privileged support, whose reactivity can be tuned by thermal activation. This study provides a qualitative and quantitative assessment of the hydroxyl groups present on the surface of γ‐Al_2_O_3_ at three different dehydroxylation temperatures. The principal [AlOH] configurations are identified and described in unprecedented detail at the molecular level. The structures were established by combining information from high‐field ^1^H and ^27^Al solid‐state NMR, IR spectroscopy and DFT calculations, as well as selective reactivity studies. Finally, the relationship between the hydroxyl structures and the molecular‐level structures of the active sites in catalytic alkane metathesis is discussed.

## Graphical abstract

The surface hydroxyl network of gamma‐Al_2_O_3_ was studied over a 300–700 °C dehydroxylation temperature range. High‐field MAS NMR, IR and DFT were harnessed to provide an unprecedented qualitative and quantitative assessment of the AlOH network, with impact on understanding of alkane conversion catalysts.

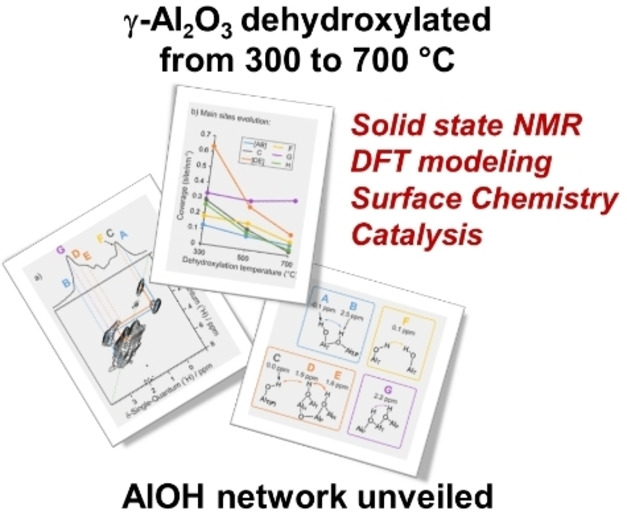

## Introduction

Transition aluminas are highly valued materials in both catalysis and adsorption technologies, finding use in a significant number of major industrial processes.^[1]^ The importance and versatility of these materials are derived, in part, from the richness of their surface chemistry, which features a combination of Lewis and Brønsted acidic and basic sites. Among the transition aluminas, γ‐Al_2_O_3_ holds a privileged position.^[2]^ The nature and quantity of reactive sites on its surface depend directly on its thermal pretreatment. More specifically, thermal processing affects the surface hydroxyl distribution and density. The characterization of hydroxyl networks on inorganic supports is a major study field.^[3]^ Many spectroscopic studies have been devoted to the investigation of these hydroxyl groups, although IR spectroscopy is by far the most widely‐used technique.^[4]^ It can be very sensitive to minor changes in local environment, which can yield a wealth of information about hydroxyl group relationships. However, spectroscopic assignments are often not straightforward, and connections to local arrangements depend largely on indirect comparisons. High‐resolution transmission electron microscopy has also been harnessed to study the surface of this material, although the relationship to results from other techniques is not straightforward.^[5]^

In principle, solid‐state NMR spectroscopy can provide much more detailed information about the nature of the surface hydroxyls and their relationships, due to the resolution of both ^1^H and ^27^Al NMR 1D spectra at high magnetic fields, as well as the power of advanced NMR methods, namely, homo‐ and heteronuclear correlations.^[6]^ Together, they offer the possibility of advancing our current state‐of‐the‐art understanding of the alumina surface. For instance, homonuclear correlations such as ^1^H‐^1^H DQSQ MAS NMR can reveal proximities between hydroxyl groups, while robust heteronuclear correlations such as ^1^H‐^27^Al *D*‐HMQC MAS NMR provide information about the nature of the Al centers associated with specific protons (e.g., coordination numbers and, potentially, quadrupolar coupling constants).^[7]^ NMR parameters of surface species (hydroxyl groups or metal‐based grafted entities) can also be determined by calculation, providing a powerful tool when characterizing inorganic supports.^[8]^ Combination of experimental NMR results and theoretical studies give rise to the field of NMR crystallography. The latter is ideally suited to the study of highly ordered materials, such as the catalytically relevant zeolites.^[9]^ In contrast, γ‐alumina has been much less studied, in part because its surface represents only a minor part of the solid and it is disordered.

To advance our understanding of these materials, we need more than empirical assignments. We must develop well‐grounded proposals that describe the precise molecular‐level arrangements of the surface hydroxyl groups.^[6]^ In this context, theoretical modeling based on molecular dynamics have been successfully adopted to understand the surface/water interphase^[10a]^ and the effect of hydration on the surface structure.[^10b^, ^10c^] DFT studies of the γ‐alumina surface^[11]^ have given us very detailed proposals about the surface structure, but there has unfortunately been relatively little comparison to experimental IR spectra, or to only 1D ^1^H or ^27^Al NMR data.^[12]^ Moreover, controversies still arise about the precise nature of the crystalline phase of the bulk structure.^[13]^ Consequently, many conclusions about the structure of the γ‐alumina surface which are derived primarily from DFT modeling lack experimental confirmation. An alternative strategy uses results from advanced experimental methods to propose structures based on robust spectroscopic data, then compares these structures with those calculated using DFT methods.

In this study, we take the latter approach to investigate γ‐alumina treated at various temperatures (300, 500 and 700 °C, designated **Al_2_O_3‐300_**, **Al_2_O_3‐500_** and **Al_2_O_3‐700_**, respectively), and propose a unifying structural model, supported by DFT calculations, for the surface hydroxyl distribution. Initially, we focus on **Al_2_O_3‐700_**, since its relatively low surface hydroxyl density results in a simpler overall surface structure. Using ^1^H‐^1^H dipolar homonuclear correlation maps and ^1^H‐^27^Al dipolar heteronuclear correlation spectra, complemented by observations of reactivity towards HCl and CO_2_, we deduce the main elements of the surface hydroxyl topology. Next, we extend the study to both **Al_2_O_3‐300_** and **Al_2_O_3‐500_**, resulting in a quantitative and qualitative assessment of the evolution of surface hydroxyl sites over this temperature range. The conclusions are strengthened by comparison of the proposed structural features to the most stable species calculated by DFT. Finally, we connect the surface hydroxyl structure to the performance of these catalytic materials in alkane metathesis, illustrating both the significance of our findings and the current limitations of our approach.

## Results and Discussion

**Figure 1:**
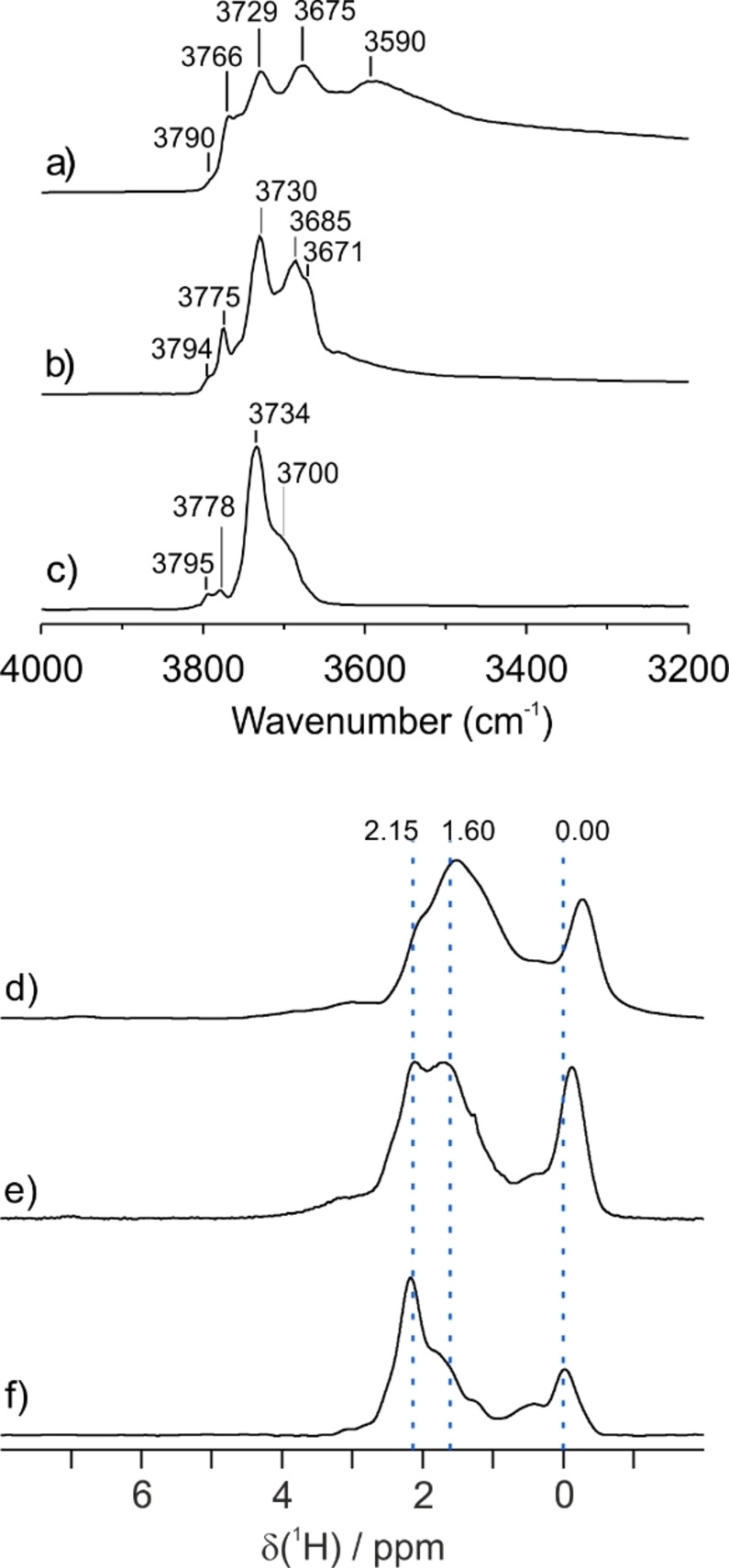
Comparison of spectra for several γ‐aluminas: DRIFTS spectra in the hydroxyl stretching region, for a) **Al_2_O_3‐300_**, b) **Al_2_O_3‐500_**, and c) **Al_2_O_3‐700_**, as well as ^1^H MAS NMR spectra (18.8 T, spinning frequency 20 kHz), for: d) **Al_2_O_3‐300_**, e) **Al_2_O_3‐500_**, and f) **Al_2_O_3‐700_**.

Typical IR spectra for **Al_2_O_3‐300_**, **Al_2_O_3‐500_** and **Al_2_O_3‐700_** are shown in Figure 1. Similar spectra, consisting of several fairly well‐defined bands, have frequently been reported in the literature. They reveal different types of hydroxyl groups (terminal and bridging, associated with different types of surface Al atoms) that coexist on the surface, in proportions that evolve depending on the severity of the thermal pretreatment. Similarly, ^1^H MAS NMR spectra of γ‐alumina consist of groups of signals in specific chemical shift (CS) regions. They are reasonably assigned to terminal hydroxyl groups, μ^1^‐OH, at ca. 0 ppm, as well as doubly‐bridging hydroxyl groups, μ^2^‐OH, between 1 and 3 ppm (Figure 1d–f). IR signals below 3590 cm^−1^, and ^1^H‐NMR signals with CS higher than 2.5 ppm, are traditionally assigned to triply‐bridging hydroxyl groups, μ^3^‐OH, without experimental evidence. This assignment was recently challenged,^[14]^ and will be discussed in more detail below.

In the IR, the precise O−H stretching frequencies shift slightly with thermal treatment,^[4]^ suggesting that the surface hydroxyl density affects ν(OH) due to proximity effects. This finding is consistent with a non‐random initial arrangement of hydroxyl groups,[^14^, ^15^] as well as a precise pattern for their evolution upon increasing dehydration. Thus at least some of the hydroxyl types appear to exist in more complex substructures. The resolution of the ^1^H MAS NMR spectra also reveals the existence of several sites within each chemical shift region, suggesting different environments for each type of hydroxyl group.

### Hydroxyl Topology of the Al_2_O_3‐700_ Surface

Of the three partially dehydroxylated aluminas, **Al_2_O_3‐700_** has simplest IR and ^1^H MAS NMR spectra, and will be considered in detail first. The IR spectrum of **Al_2_O_3‐700_**, Figure 1c, displays four main bands in the O−H stretching region, at 3795, 3778, 3734 and 3700 cm^−1^. It is widely accepted that terminal hydroxyl groups, μ^1^‐OH, give rise to the two highest frequencies, whereas doubly‐bridging hydroxyl groups, μ^2^‐OH, give rise to the two lower frequency bands. The ^1^H MAS NMR spectrum of **Al_2_O_3‐700_**, recorded at a very high magnetic field (18.8 T, Figure 1f), is remarkably well resolved, featuring multiple signals between 3 and −0.5 ppm. CS values from 1 to −0.5 ppm are typically assigned to various types of μ^1^‐OH protons, while CS values from 3 to ca. 1.2 ppm are assigned to different kinds of μ^2^‐OH protons.

**Figure 2:**
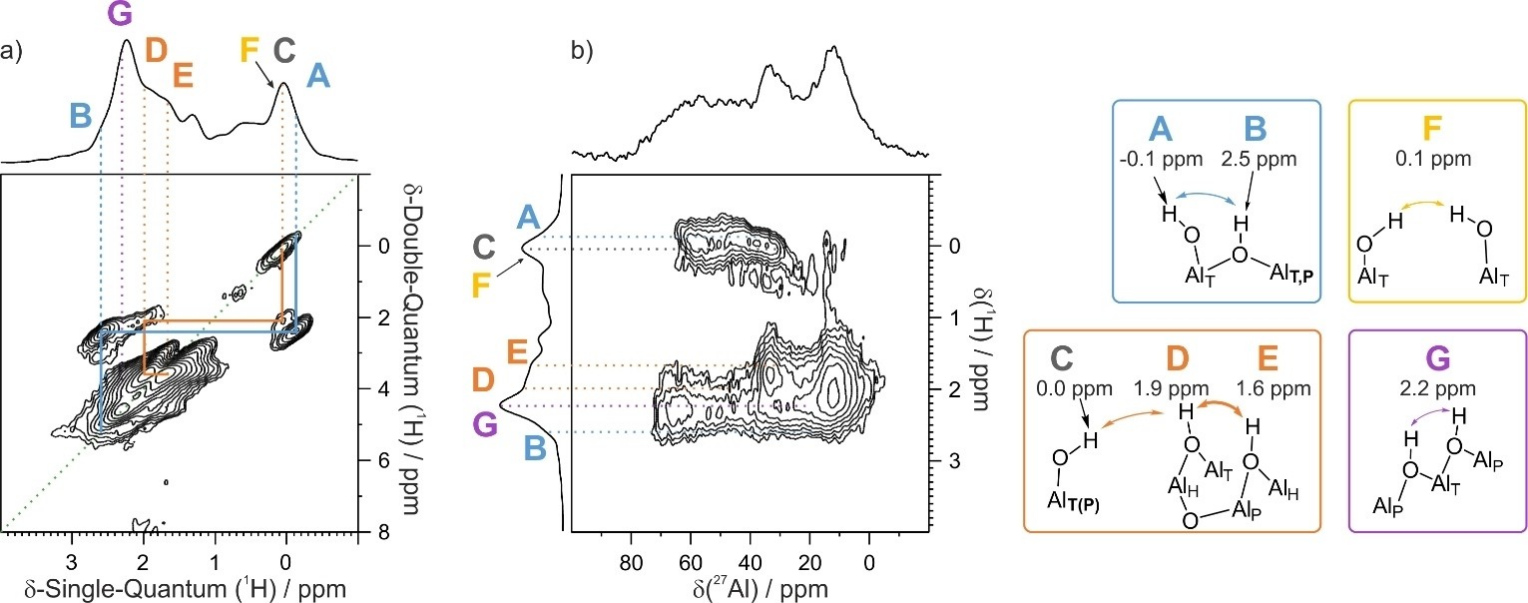
a) ^1^H‐^1^H DQSQ MAS NMR spectrum, and b) ^1^H‐^27^Al D‐HMQC MAS NMR spectrum, for **Al_2_O_3‐700_** (18.8 T, spinning speed 20 kHz), and the hydroxyl environments deduced from the correlations, with their associated ^1^H‐NMR chemical shifts.

The ^1^H‐^1^H DQSQ MAS NMR spectrum (Figure 2a) provides further information on which to base more detailed structural assignments. It shows several well‐resolved off‐diagonal interactions, reflecting proximity between protons with distinct chemical shifts. In total, there are three major off‐diagonal correlations in the 2D spectrum, as discussed below, indicated by the blue and orange lines. Two of them show proximity between a μ^1^‐OH site, either **A** (−0.11 ppm) or **C** (0.03 ppm), to one of the μ^2^‐OH sites, **B** (2.5 ppm) or **D** (1.9 ppm), respectively. Sites **D** and **E** (1.6 ppm) are the third pair; they belong to a group of μ^2^‐OH sites whose proximity we reported in a previous study.^[6]^ In the case of the **A**–**B** pair, the strong cross‐peak intensities relative to their weak intensities in the 1D MAS NMR spectrum indicate that the distance between these protons is very short. We propose that they are bonded to the same Al center, as illustrated in Figure 2. The high intensity of the correlations for the **D**–**E** pair indicates that the corresponding hydroxyls are also closely co‐located. The ^1^H‐^1^H DQSQ MAS NMR spectrum in Figure 2a shows several auto‐correlations, located on the diagonal of the spectrum, reflecting proximity between protons with similar CS values (i.e., non‐isolated, similar protons). Two of these correlations arise from the signals labeled **F** (CS 0.08 ppm, therefore a terminal hydroxyl) and **G** (CS 2.23 ppm, therefore a doubly‐bridging hydroxyl).

The coordination number/geometry of the Al centers bearing different types of hydroxyl groups can be deduced from the ^1^H‐^27^Al *D*‐HMQC MAS NMR spectrum (Figure 2b). The terminal hydroxyl groups (μ^1^‐OH, ^1^H CS ca. 0 ppm) are predominantly in close proximity to tetracoordinated aluminum sites, Al_T_, at ^27^Al CS ca. 70 ppm. As previously reported,^[16]^ these Al_T_ sites exhibit large quadrupolar coupling constants, up to 12 MHz based on best‐fit simulations. Terminal hydroxyls associated with penta‐coordinated aluminum sites, Al_P_, at ^27^Al CS ca. 40 ppm are also present, though to a lesser extent. There is only a weak intensity correlation between μ^1^‐OH and hexacoordinated aluminum sites, Al_H_ (^27^Al CS 20–0 ppm).

Among the doubly‐bridging hydroxyl groups (μ^2^‐OH, ^1^H CS 1–3 ppm), sites **D** and **E** are mostly correlated to ^27^Al sites with CS values below 40 ppm, i.e., Al_p_ and Al_H_ sites. Within this block, site **D** is closest to site **C**, a terminal hydroxyl associated with Al_T_ or, to a lesser extent, Al_P_. The auto‐correlated signals **F** and **G** show proximity with Al_T_ and/or Al_P_ sites. These spectroscopic and structural relationships are summarized in Figure 2, representing the most probable surface hydroxyl sites on **Al_2_O_3‐700_**. The proposed structural motifs are also based on insights from DFT modeling, to be presented in a dedicated section below. Noteworthy, additional sites at 0.5 and 1.3 ppm, denoted as **X1** and **X2** in Supporting Information (Figure S4), still evade assignments at the current stage. They display no significant signal in ^1^H‐^1^H DQSQ or in ^1^H‐^27^Al *D*‐HMQC MAS NMR spectra.

**Figure 3:**
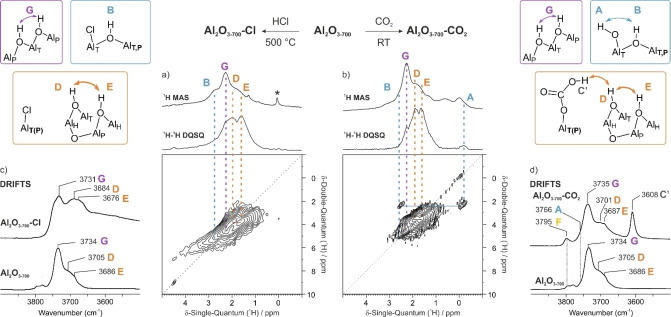
^1^H MAS NMR and ^1^H ‐^1^H DQSQ MAS NMR spectra of a) **Al_2_O_3‐700_‐Cl**, and b) **Al_2_O_3‐700_‐CO_2_** (18.8 T, spinning speed 20 kHz; * impurity, not present in the 2D spectrum), and their corresponding IR spectra (c, d, arbitrary intensity scale). Proposed structures for various hydroxyl‐bearing Al sites present after reaction are also shown.

To refine these assignments, we resorted to selective reactions of the surface hydroxyl groups. The first such reaction involves partial surface chlorination using a previously described method.^[17]^ **Al_2_O_3‐700_** was treated with HCl_(g)_ at 700 °C, followed by evacuation at the same temperature, to afford **Al_2_O_3‐700_‐Cl**. This procedure is reported to result in complete reaction of the terminal hydroxyl groups. The ^1^H MAS NMR spectrum features signals in the 3–1 ppm range but no meaningful signals in the 1–0 ppm range (Figure 3a), confirming the selectivity of chlorination at the terminal hydroxyls (This finding does not preclude partial chlorination of μ^2^‐OH groups). The substitution of terminal hydroxyls by chlorine results in moderate shifts of all isotropic CS values for **B**, **D**, **E** and **G** protons by +0.03–0.20 ppm, from 2.50, 1.90, 1.65 and 2.23 in **Al_2_O_3‐700_** to 2.70, 1.95, 1.60 and 2.26 ppm, respectively, in **Al_2_O_3‐700_‐Cl**. These shifts are most likely the result of changes in electrostatic interactions.

The chlorinated material was probed by ^1^H‐^1^H DQSQ MAS NMR (Figure 3a). Once again, the most prominent feature on the correlation map associates the two doubly‐bridging hydroxyls **D** and **E**, at 1.95 and 1.60 ppm, respectively. An on‐diagonal correlation arises due to **G**‐type bridging hydroxyls, which give rise to the most prominent ^1^H MAS NMR signal, at 2.26 ppm. Another ^1^H MAS NMR signal appears as a shoulder at ca. 2.7 ppm and is assigned to a **B**‐type bridging hydroxyl, by comparison to the spectrum of **Al_2_O_3‐700_**. Since the terminal hydroxyls are no longer present, **B** sites do not give rise to an off‐diagonal (**A**–**B**) cross‐correlation, as was observed in the ^1^H‐^1^H DQSQ MAS NMR spectrum of pristine **Al_2_O_3‐700_** (Figure 2a). The 2D spectrum of Figure 3a also reveals an off‐diagonal signal covering a wide CS range, extending beyond 3 ppm (see below).

The DRIFTS spectrum of **Al_2_O_3‐700_‐Cl** (Figure 3c) confirms the absence of μ^1^‐OH groups (formerly absorbing at 3778 and 3794 cm^−1^) after chlorination, in line with the ^1^H MAS NMR observations. Three broad bands remain at 3731, 3684 and 3676 cm^−1^, in the frequency range typical of μ^2^‐OH groups. We propose that **G**‐type hydroxyls are responsible for the most intense band at 3731 cm^−1^, with minimal change in frequency upon chlorination (3734 cm^−1^ in pristine **Al_2_O_3‐700_**). In contrast, the bands assigned to bridging hydroxyl groups **D** and **E**, at 3705 and 3686 cm^−1^, respectively, shift much more upon chlorination. More support for these assignments will be presented below, when we address the evolution of the hydroxyl groups with increasing dehydroxylation of the alumina surface.

In a second probe reaction, **Al_2_O_3‐700_** was exposed to CO_2(g)_ at room temperature, affording **Al_2_O_3‐700_‐CO_2_**. The reaction with CO_2_ also occurs preferentially at the terminal hydroxyl sites,[^6^, ^18^] resulting in the disappearance of the weak band at 3778 cm^−1^ in **Al_2_O_3‐700_**, and the appearance of an intense new band (ν_OH_=3608 cm^−1^) assigned to the carbonate group (**C′**) formed by the reaction of acidic CO_2_ with basic site **C**. In the ^1^H‐^1^H DQSQ MAS NMR spectrum, the **C**–**D** correlation is absent (Figure 3b), while a weak correlation between **C′** (6.0 ppm) and **D** is visible (Figure S2). Increased mobility of the **C′** proton compared to that of μ^1^‐OH **C** is most probably at the origin of the observed low intensity of the cross‐peaks. In addition, partial consumption of site **A** is evidenced by the weaker off‐diagonal signal for **Al_2_O_3‐700_‐CO_2_** compared to **Al_2_O_3‐700_**. Since site **F** is still present and gives rise to a self‐correlation on the DQSQ spectrum, the band at 3795 cm^−1^ in the IR spectrum of **Al_2_O_3‐700_‐CO_2_** is assigned to this type of terminal hydroxyl group.

### Effect of Thermal Treatment on the Distribution of Surface Hydroxyl Groups

The decrease in surface OH density with increasing annealing temperature has been quantified in many studies, using methods such as chemical titration, TGA, and ^1^H‐NMR spectroscopy.[^12^, ^19^] In this study, the values measured by chemical titration with Al*i*Bu_3_ for **Al_2_O_3‐300_**, **Al_2_O_3‐500_** and **Al_2_O_3‐700_** are 3.0, 1.8 and 1.1 OH nm^−2^, respectively, and are in line with reported values. According to both IR and ^1^H MAS NMR (Figure 1), the nature of the surface OH groups is also affected by the extent of dehydroxylation. Using our detailed NMR approach, we undertook a combined qualitative and quantitative analysis of the hydroxyl networks in **Al_2_O_3‐300_**, **Al_2_O_3‐500_** and **Al_2_O_3‐700_**, based principally on comparison of their ^1^H‐^1^H DQSQ MAS NMR spectra, as well as best‐fit simulations of their ^1^H MAS NMR spectra.

In general, all of the principal types of hydroxyl groups identified in **Al_2_O_3‐700_** are still present in aluminas dehydroxylated at lower temperatures (500 and 300 °C), but in different relative proportions (Figure S3). The latter observation suggests selective dehydroxylation at the molecular scale. As expected, the abundance of interacting hydroxyl groups is much higher in the aluminas with higher hydroxyl densities (see below). This finding suggests that the global connectivity map is maintained throughout the dehydroxylation process, even as some clusters of hydroxyl sites are more affected than others. Interestingly, there is a global increase in ^1^H chemical shift for each site with increasing dehydroxylation temperature (i.e., with decreasing OH density). The CS variation can be up to 0.3 ppm, e.g., in the case of site **C** (see Table S1).

**Figure 4:**
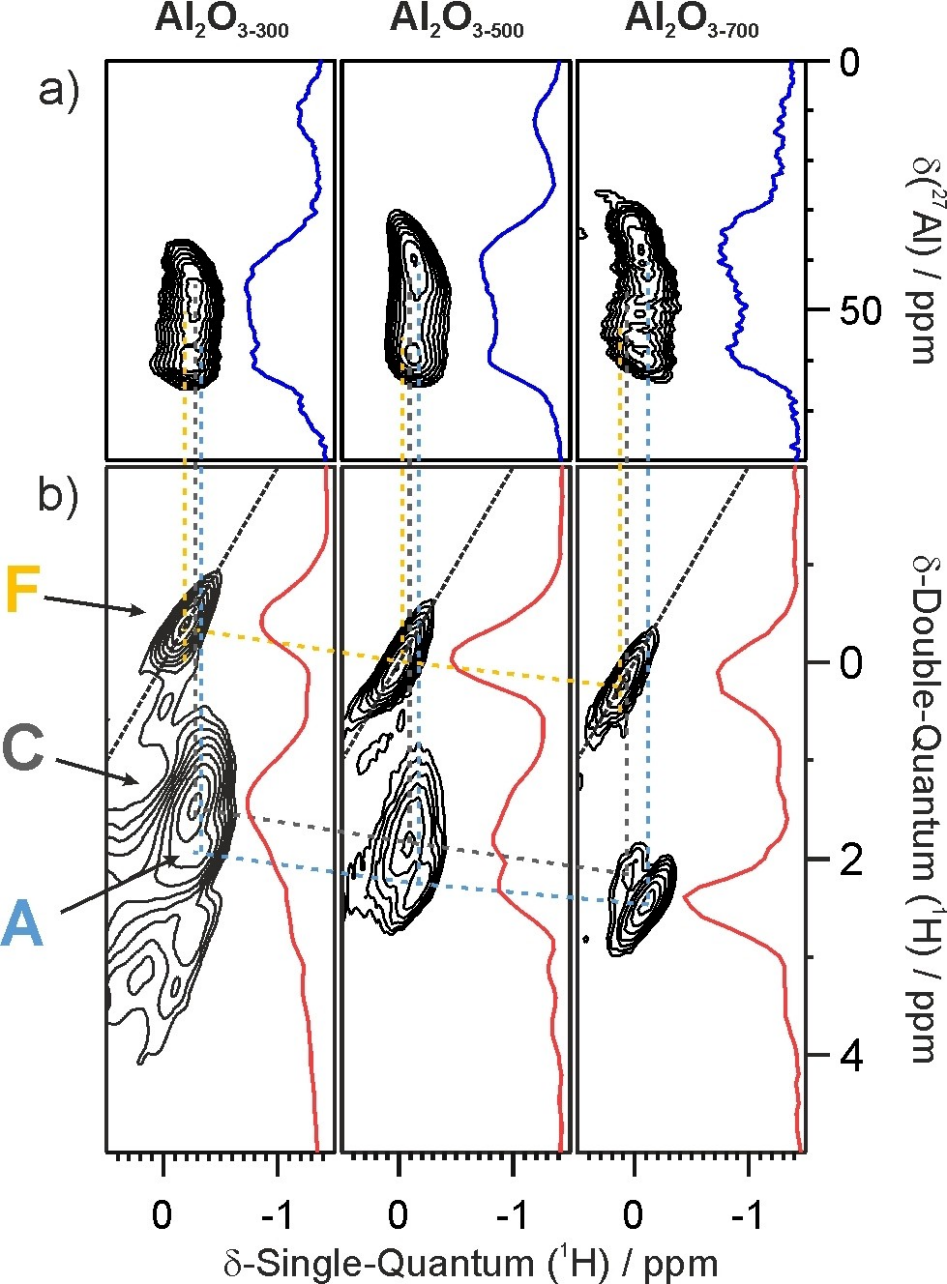
Comparison in the region of the terminal hydroxyl group signals, of a) ^1^H‐^1^H DQ‐SQ NMR spectra, and b) ^1^H‐^27^Al D‐HMQC MAS NMR spectra (18.8 T, spinning speed 20 kHz) for **Al_2_O_3‐300_**, **Al_2_O_3‐500_** and **Al_2_O_3‐700_**, as well as the corresponding projections in the indirect dimension (red and blue traces).

For the terminal hydroxyl sites, the most striking finding from the ^1^H‐^1^H DQSQ MAS NMR spectra concerns site **C** (^1^H CS −0.1 ppm). Its cross‐peak intensity (correlating with site **D**) decreases steadily with increasing dehydroxylation temperature, until **C** becomes a minor species at 700 °C (Figure 4). The other terminal hydroxyl sites **A** and **F** are only slightly affected by more extensive dehydroxylation. As for the bridging hydroxyls, the quantitative ^1^H 1D MAS NMR spectra show a continuous decrease in intensity for both **D** and **E** signals from 300 to 700 °C (Figure 1). Nevertheless, these sites give rise to the most intense signals in the DQSQ spectra, regardless of the dehydroxylation temperature, due to their very strong dipolar coupling. For site **G** (^1^H CS 2.23 ppm), the signal is not visible in the DQSQ spectra after dehydroxylation at 300 °C, probably due to masking by the strong signals of the dominant **D** and **E** sites. However, it is clearly present in the 1D ^1^H MAS spectra as a shoulder at ca. 2.2 ppm.

Finally, the aluminas dehydroxylated at 300 and 500 °C show a strong off‐diagonal pattern at CS values above 2.5 ppm, due to a large dipolar coupling that associates H‐bonded hydroxyl groups of type **H** with specific μ^2^‐OH sites (both **D** and **E** at 300 °C, and mainly **D** at 500 °C). Interestingly, the ^1^H intensity at CS values above 3 ppm is magnified in the DQSQ spectra of **Al_2_O_3‐300_** and **Al_2_O_3‐500_**, indicating short distances between site **H** and sites **D** or **C**. However, the small relative intensity in the ^1^H MAS NMR spectra indicates that **H** sites are present in only minor quantity.

The relative intensities of the two main cross‐peaks (**A** and **C**) and the on‐diagonal peak (**F**) in the ^1^H‐^1^H DQSQ MAS NMR spectra evolve as the dehydroxylation temperature increases from 300 to 700 °C (Figures 4 and S5). In particular, the contribution of site **C** decreases. Concomitantly, the ^27^Al signal in the ^1^H‐^27^Al *D*‐HMQC‐NMR spectrum for sites bearing terminal hydroxyl groups (Figure 4a, see also Figure S6 for the full spectra) broadens with increasing dehydroxylation temperature, consistent with the variation in relative abundance of the **A**, **C** and **F** sites and/or with increased quadrupolar coupling due to more severe structural distortion. **A** or **F** sites may contain either Al_T_ or Al_P_ centers, although the poor resolution in the ^1^H dimension precludes a more precise assignment at this time. The low resolution in the HMQC experiment is due to the similarity in ^1^H‐NMR chemical shifts and large ^27^Al quadrupolar coupling constants which cause signal overlap. At 18.8 T, the interpretation of these spectra has reached its current technical limit, although we know that the most abundant type of terminal hydroxyl group is associated with Al_T_, and the next most abundant with Al_P_.

**Figure 5:**
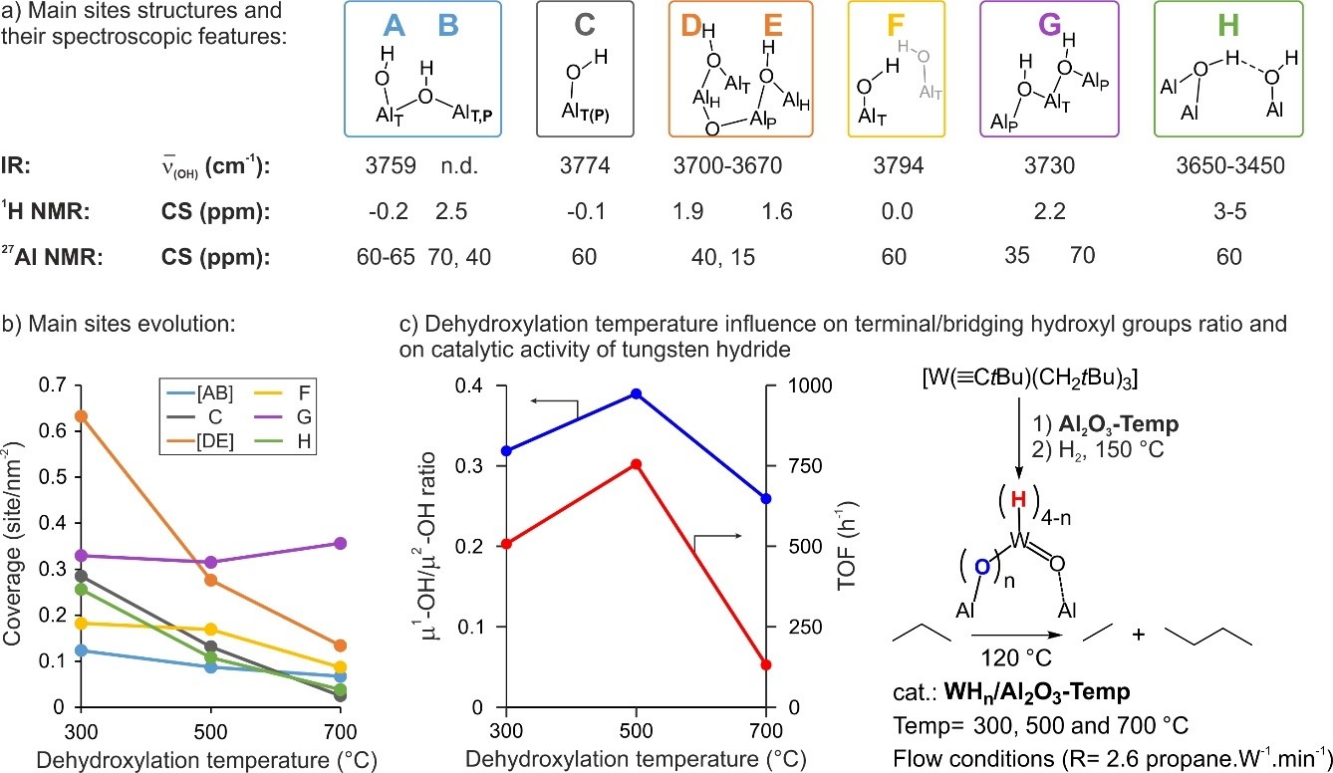
a) Structures of the main hydroxyl sites and their spectroscopic features (values shown for **Al_2_O_3‐500_**), b) evolution of the coverages of the main hydroxyl sites, and c) relationship between the ratio of terminal to bridging hydroxyls (which varies with thermal pretreatment of alumina) and catalytic performance in propane metathesis, for tungsten hydride catalysts supported on **Al_2_O_3‐300_**, **Al_2_O_3‐500_** and **Al_2_O_3‐700_**.

Quantitative information can be extracted from best‐fit simulations of the ^1^H MAS NMR spectra, based on the DQSQ information (Figure S4, Table S2). The combination of the 1D spectra and the off‐diagonal peaks provides a complete set of CS values, even for sites with strongly overlapping signals (e.g., in the μ^1^‐OH region). The result is shown in Figure 5b, where the relative proportions determined from the best‐fit simulations are weighted by the overall OH surface density. These unprecedented data provide individual surface densities for each type of surface hydroxyl group over the range of dehydroxylation temperatures considered here. The sites most affected by temperature, besides the **H** sites (as is already apparent from ^1^H MAS NMR spectra, see above), are related to the **C**–**D**–**E** cluster. A decrease in abundance for sites **A**–**B** and **F** is observed for dehydroxylation temperatures above 500 °C. Interestingly, the behavior of site **G** (a clustered μ^2^‐OH site, as evidenced by its autocorrelation in the DQSQ experiment) is peculiar: its intensity seems unaffected throughout the temperature range.

Regarding the lowest‐field signals (**H** sites, with ^1^H CS above 2.7 ppm) in the ^1^H‐NMR spectra of **Al_2_O_3‐300_** and **Al_2_O_3‐500_**, we concur with the recent assignment of Lesage and Raybaud,^[14]^ namely that these are not triply‐bridging hydroxyl groups (μ^3^‐OH), contrary to what we and others proposed before. Most likely, these signals stem from interacting (H‐bonded) hydroxyl groups. This revised assignment is consistent with the higher hydroxyl density and therefore higher likelihood for H‐bonding configurations on **Al_2_O_3‐300_** and **Al_2_O_3‐500_**, relative to **Al_2_O_3‐700_**. The influence of H‐bonding on ^1^H‐NMR chemical shifts was reported previously,[^12^, ^20^] *viz*., stronger H‐bonding induces higher chemical shifts, resulting in values up to about 6 ppm.

Using these findings, we can further refine our IR spectroscopic assignments for the ν(OH) modes of the terminal hydroxyl groups. The band at 3778 cm^−1^ is assigned to site **C** based on the reaction of **Al_2_O_3‐700_** with CO_2_ (see above). It shifts from 3769 cm^−1^ at 300 °C to 3778 cm^−1^ at 700 °C. Since **F** is always a significant fraction of the terminal hydroxyl groups, and considering the evolution of the IR spectra in the region 3800–3760 cm^−1^ (Figure S7), we propose that **F** sites give rise to the band at 3795 cm^−1^ (on **Al_2_O_3‐700_**). The band at ca. 3760 cm^−1^, which is the most intense signal in the region for **Al_2_O_3‐300_** but a minor contribution for **Al_2_O_3‐700_**, is assigned to **A** sites.

### DFT Modeling and Comparison to Experimental Data

#### DFT Surface Model

In order to support our experimental assignments, we resorted to modelling the surface of the alumina with the simplest spectrum, namely, **Al_2_O_3‐700_**. The γ‐alumina bulk model used in this study is taken from the theoretical investigations of Digne et al.^[11a]^ The structure (shown in Figure S9) consists of a sub‐lattice (fcc) of oxide ions with octahedral and tetrahedral interstices that accommodate aluminum ions. Recent experimental data for γ‐alumina indicate that, in a spinel‐type indexing, the (110) surface predominates even though it undergoes a significant reconstruction, forming nanoscale (111) Al_2_O_3_ facets.^[5]^ According to Digne et al., the (100) face is completely dehydrated after treatment at 423 °C and above (i.e., for **Al_2_O_3‐500_** and **Al_2_O_3‐700_**).^[11a]^ Thus, our analysis will focus on the (110) surface since it represents the principal exposed crystallographic surface, and thus accounts for most of the experimentally observable surface groups.

Truncation of the bulk structure to generate the (110) surface results in surface Al atoms with either trigonal symmetry (Al_Tri_, derived from tetrahedral Al atoms in the bulk), or pseudo‐tetrahedral symmetry (Al_T_, arising from octahedral atoms in the bulk). Surface O ions have either μ^2^‐O or μ^3^‐O coordination geometries. The latter are bound to one Al_Tri_ and one Al_T_ surface ion, as well as one Al_H_ ion in the bulk, while the former are bound to an Al_T_ surface ion and an octahedral Al_H_ bulk ion (Figure S9).

According to the experimental data, the surface hydroxyl density for γ‐alumina annealed at 700 °C is ca. 1.1 OH nm^−2^. From the simulations, adsorption of one water molecule on the modelled surface leads to a hydroxyl density of 0.7 OH nm^−2^, while adsorption of two water molecules gives a hydroxyl density of 1.5 OH nm^−2^. Here we consider both cases, i.e., one and two absorbed water molecules, and investigate the most stable hydroxyl configurations that result.

#### Adsorption of One Water Molecule

**Figure 6:**
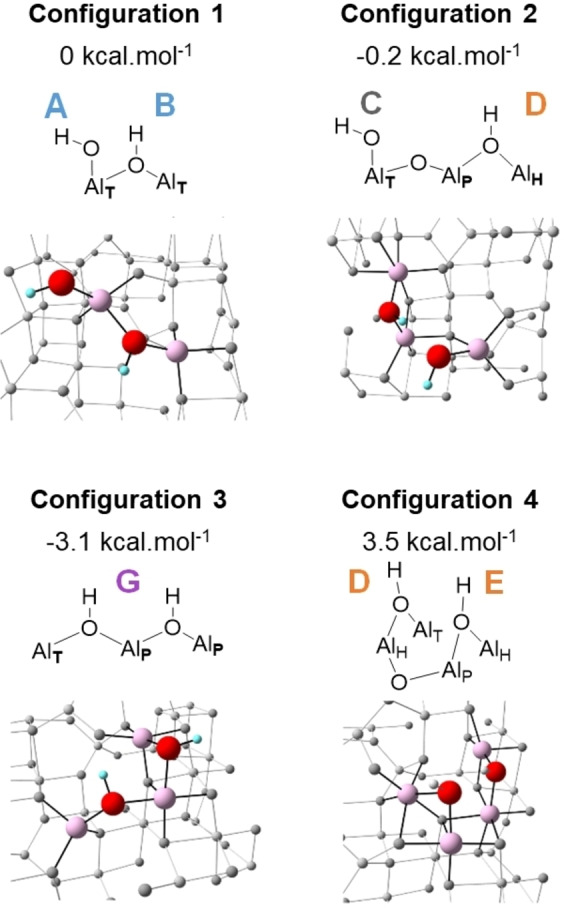
Calculated hydroxyl group configurations and their relative energies, for the partially hydroxylated (110) surface of γ‐alumina with *θ*=0.7 OH nm^−2^.

Water can, in principle, adsorb on either an Al_Tri_ site or an Al_T_ surface site, since both are coordinatively unsaturated (Figure S9). Once the water molecule adsorbs, one proton moves towards a surface oxygen (either μ^2^‐O or μ^3^‐O), resulting in two proximal hydroxyl groups. Water adsorption on an Al_Tri_ surface site generates a Al_T_‐μ^1^‐OH species. In this case, the other proton migrates onto either a vicinal μ^2^‐O site, producing a bridging hydroxyl (Al_T_‐μ_2_‐OH‐Al_T_, corresponding to Configuration **1** in Figure 6), or onto an adjacent μ^3^‐O site, producing a different bridging hydroxyl (Al_H_‐μ^2^‐OH‐Al_P_, corresponding to Configuration **2** in Figure 6). Alternatively, the water molecule can adsorb between a pair of unsaturated Al_T_ surface sites, forming a bridge between them. The proton migrates preferentially onto either the vicinal μ^2^‐O site, yielding Configuration **3**, or onto a vicinal μ^3^‐O site producing Configuration **4** (Figure 6). When protons migrate onto μ^3^‐O sites, their geometrical environment is strongly distorted, and they always rearrange to form μ^2^‐OH surface groups. Thus, μ^3^‐OH groups are unlikely to persist on the surface.

Even though the geometrical arrangements of the surface hydroxyls allow formation of hydrogen bonds, our calculations are performed at 0 K and, therefore, do not take into account thermal effects which can weaken (or destroy) hydrogen bonding.

#### Adsorption of Two Proximal Water Molecules

All possible structures involving the adsorption of two water molecules can be derived from a combination of the four configurations previously described. In order to provide a comparison with the experimental findings, the combination of configurations **1** and **4** (Configuration 1/4, Figure S10) will be selected to support our discussion in the next section.

#### Convergence with Experimental Data

Based on these calculated structures, a direct comparison to our spectroscopic data is now possible. To‐date, the fairly extensive theoretical studies of the surface hydroxyl network have not been supported by much more than simple ^1^H MAS NMR or IR data. Here, the availability of high‐resolution NMR and bidimensional NMR correlations provides a unique opportunity to compare our theoretical models with precise structural evidence at the molecular level. Indeed, several features that appear in the ^1^H‐^1^H DQSQ MAS and ^1^H‐^27^Al D‐HMQC MAS NMR spectra of **Al_2_O_3‐700_** can be confidently assigned to specific sets of hydroxyl sites that were proposed as a result of the DFT calculations (see below). Considering the complexity of alumina surface chemistry, both experimentally and theoretically, we do not expect the model to coincide perfectly with the experimental data, especially with respect to the coordination numbers of the Al centers. However, geometric patterns reflecting the most stable arrangements may emerge.

The first salient point is the close resemblance between DFT‐calculated configuration **1** and the **A**–**B** set. The calculated distance between the hydrogen atoms of the Al_T_‐μ^1^‐OH and Al_T_‐μ^2^‐OH‐Al_T_ hydroxyl groups is ca. 3.7 Å, with a calculated dipolar coupling of 2.5 kHz. The latter is in line with the magnitude required to account for the off‐diagonal signal in the ^1^H‐^1^H DQSQ MAS NMR arising from protons on sites **A** and **B**. Furthermore, both Al sites are tetra‐coordinated, consistent with conclusions arising from the ^1^H‐^27^Al D‐HMQC MAS NMR data. There is a similar convergence between calculated Configuration **2** and the **C**–**D** set (see below). It involves the same Al_T_‐μ^1^‐OH group and a vicinal Al_H_‐μ^2^‐OH‐Al_P_ bridging hydroxyl.

Using the same type of reasoning, the auto‐correlating **G** signal is best represented by Configuration **3**. According to the *D*‐HMQC data, these OH groups are located on Al_T_ and Al_P_ sites, and the strong dipolar coupling is consistent with the calculated value of 13 kHz (from a distance of 2.08 Å). The two doubly‐bridging hydroxyls share a common Al center, and their quasi‐linear arrangement may account for the difficulty in their removal by dehydroxylation (H_2_O release), based on geometrical constraints. Moreover, it is the most stable configuration among all those shown in Figure 6. This finding is in line with the stability of the **G** sites as described above, since their surface concentration is essentially constant over our temperature range (Figure 5b). However, as the real material is a mixture of crystal facets, some of which (that may contain **G**) may dehydroxylate more readily than others.

Similarly, the structural arrangement of configuration **4** can be related to the two doubly‐bridging hydroxyl groups in the **D**–**E** block. The experimentally‐observed strong dipolar coupling between **D** and **E** protons can be related to the coupling between hydroxyl groups co‐located in a side‐by‐side geometry, and associated mostly with penta‐ and hexacoordinated Al centers. In DFT‐calculated configuration **4**, they are linked by one μ^2^‐ and one μ^3^‐O atom as Al_H_‐μ^2^‐OH‐Al_P_ and Al_P_‐μ^2^‐OH‐Al_H_ species, with a calculated distance between the hydrogen atoms of 2.4 Å, and a strong dipolar coupling of 8.0 kHz. This local configuration, in which the two hydroxyl groups are close to one another, may facilitate water elimination upon thermal treatment. Indeed, Figure 5b shows that the **D**–**E** block undergoes the earliest decrease in concentration of any hydroxyl pair as the dehydroxylation temperature is raised from 300 to 700 °C.

Finally, the combination of configurations **1** and **4** results in a chain of hydroxyl groups which includes one μ^1^‐OH and two μ^2^‐OH sites. They are consistent with the **C**–**D**–**E** cluster identified from dipolar interactions (Figure 2). The calculated dipolar coupling for the **C**–**D** interaction, 1.7 kHz, is significantly lower than that of the **D**–**E** interaction, in line with the experimental data.

DFT calculations can also evaluate the quadrupolar coupling constants (C_Q_) associated with different Al surface sites. Surface tetra‐coordinated Al sites (Al_T_, involved in μ^1^‐OH bonding) have C_Q_ values in the range 18–23 MHz, depending on the local structure and chemical environment. Penta‐coordinate Al sites (Al_P_, involved in μ^2^‐OH bonding) show smaller C_Q_ values in the range 10–15 MHz, while hexa‐coordinate Al sites (Al_H_, also involved in μ^2^‐OH bonding) have the smallest C_Q_ values, in the range 3.9–4.3 MHz. Although these values are higher than those obtained experimentally, the trend is as expected based on Al coordination number.^[6]^ Thus, the experimental evidence combined with theoretical studies lead us to propose the structure–property correlations in Figure 5a for the principal surface hydroxyl sites and their characteristic spectroscopic signatures (IR, ^1^H and ^27^Al NMR).

The identification of several discrete hydroxyl groupings on the γ‐alumina surface also raises questions about their location on the solid particles. For example, the **C** sites show enhanced reactivity towards various reagents,^[4]^ and may play a specific role in catalytic activity.^[21]^ Busca proposed that enhanced reactivity is due to the presence of hydroxyls on edges.^[1]^ This explanation implies that the crystallinity and morphology of γ‐alumina (which depend on its preparative and thermal history) impact the surface chemistry. Independently, in a thorough study combining preparation, characterization, and reactivity, Kwak et al. demonstrated correlations between IR band intensities and the proportions of individual facets.^[22]^ This study led to a proposed relationship between the abundance of site **C** (along with sites **D** and **E**, in agreement with our assignments) and the proportion of the surface comprised of (100) facets. In addition, our DFT investigations failed to identify a suitable structure for site **F**, when focusing on the (110) facet. Combining such an approach with advanced NMR methods will likely add significant value in the future.

In addition to considerations of hydroxyl site accessibility due to their location on edges or on different facets, molecular‐scale details of the relationships between hydroxyl groups and Lewis acid sites is also a fruitful area for further investigation. Just as some terminal hydroxyl sites (notably, **A** and **C**) are located near bridging hydroxyls of higher Bronsted acidity, which may have an impact on their (catalytic) activity, proximity of Lewis acid centers is likely to affect the properties of hydroxyl groups. This idea is featured in an IR study of γ‐Al_2_O_3_,^[23]^ as well as in our previous investigation of alumina‐supported Re catalysts for olefin metathesis.^[24]^

### Relevance to Catalysis

To link the nature and topology of the surface hydroxyls with the properties of alumina‐based catalysts, we selected examples from surface organometallic chemistry, where detailed knowledge of the reactive surface sites and of the resulting grafted sites is the basis for a rational approach to active site design. For example, supported tungsten hydrides grafted onto alumina were shown to be remarkably active in the catalytic conversions of alkanes and alkenes.^[25]^ The active sites were prepared by grafting [W(≡C*t*Bu)(CH_2_ *t*Bu)_3_] onto γ‐Al_2_O_3_, followed by hydrogenolysis of the hydrocarbyl ligands.

The ability of tungsten hydrides supported on **Al_2_O_3‐300_**, **Al_2_O_3‐500_** and **Al_2_O_3‐700_** (named **WH_300_**, **WH_500_** and **WH_700_**, respectively) to catalyze propane metathesis was probed under flow conditions (2.6 mol_propane_ mol_W_^−1^ min^−1^). The turn‐over frequency at a time‐on‐stream of 2 h (Figure 5c) show that the activity follows a volcano curve: **WH_500_** is the most active catalyst, followed by **WH_300_**, then **WH_700_**. The origin of this behavior must lie in the surface chemistry of alumina.

The first step in the synthesis of the tungsten hydrides is the grafting of [W(≡C*t*Bu)(CH_2_ *t*Bu)_3_] onto a surface hydroxyl, affording [(Al−O)[W(≡C*t*Bu)(CH_2_ *t*Bu)_2_]. According to DFT calculations, this step occurs preferentially on terminal hydroxyl sites.^[26]^ The nature and arrangement of μ^1^‐OH groups on alumina, over the temperature range used for its thermal pretreatment, is described above. Neighboring bridging hydroxyl sites can also interact with these supported organometallic species. We observe that the activity pattern in propane metathesis bears a striking resemblance to the trend in the μ^1^‐OH/μ^2^‐OH ratio: namely, a larger share of terminal hydroxyl groups results in higher catalytic efficiency.

Furthermore, the evolution of hydroxyl site coverages (specifically, that of sites **A**, **B** and **F**) shown in Figure 5b suggests a variable influence on catalysis (and by inference, on active site structure). Most likely, subtle local changes (e.g., in the second coordination sphere of the active site) control the site‐specific catalytic activity. For example, site **C** cannot be responsible for the generation of the most active tungsten hydride species, since its relative coverage decreases from **Al_2_O_3‐300_** to **Al_2_O_3‐500_** while the activity increases. The major terminal hydroxyl groups on **Al_2_O_3‐500_** are associated with the paired site **F**, which may be key in generating the most active hydrides. This hypothesis is based on evidence that the other terminal OH groups (**C** and **A**) exist in proximity to acidic bridging hydroxyls, and this local configuration could be the origin of catalyst deactivation pathways, or of side‐reactions during catalysis, involving this acidic hydroxyl. On the other hand, recent theoretical studies on the nature of the active sites formed by hydrogenolysis of [(Al−O)[W(≡C*t*Bu)(CH_2_ *t*Bu)_2_] suggest that the real structure is [(Al−O)_*n*_[WO(H)_4−*n*_], in which an oxygen extracted from the alumina support serves to generate the W=O moiety.^[27]^ Calculations show that the participation of neighboring (bridging) hydroxyl groups is essential for this transformation. It may well be that a distinct active structure is formed in the absence of neighbouring OH groups, with superior catalytic performances.

These insights suggest the need for more of the advanced theoretical and experimental studies capable of revealing relationships between the local structures of the alumina grafting sites and the resulting active sites, with implications for catalytic performance and catalyst design.

## Conclusion

A combination of spectroscopy, reactivity, and theoretical studies has provided unprecedented insight, both qualitative and quantitative, on the surface hydroxyl groups of γ‐Al_2_O_3_, across a range of widely used dehydroxylation temperatures. In particular, high‐field homo‐ and heteronuclear MAS NMR experiments generate evidence for a new model of the surface hydroxyl network of alumina. Supported by DFT calculations, it results in new assignments for the IR spectrum. In an example of the potential of this approach to provide molecular‐level structural information relevant to catalysis, specific AlOH features are correlated with the performance of an alumina‐supported catalyst for alkane metathesis.

An important feature of the combined experimental and theoretical studies reported here is the convergence of evidence for several of the main surface hydroxyl patterns. These relationships advance our current understanding of the γ‐alumina surface significantly. Considering the importance of aluminas in many major industrial processes and applications, there is still a great need for further understanding of its surface structure, which will undoubtedly benefit from additional modeling of bulk, edge, and facet structures, from advanced Al_2_O_3_ material designs, as well as from additional characterization techniques.

## Conflict of interest

The authors declare no conflict of interest.

1

## Supporting information

As a service to our authors and readers, this journal provides supporting information supplied by the authors. Such materials are peer reviewed and may be re‐organized for online delivery, but are not copy‐edited or typeset. Technical support issues arising from supporting information (other than missing files) should be addressed to the authors.

Supporting Information

## Data Availability

The data that support the findings of this study are available from the corresponding author upon reasonable request.
