# Supplementary material for: High‐Field NMR, Reactivity, and DFT Modeling Reveal the γ‐Al2O3 Surface Hydroxyl Network
Source: Angew Chem Int Ed Engl. 2022 Aug 3;61(37):e202207316. doi: 10.1002/anie.202207316 (PMC9541507; doi:10.1002/anie.202207316)
Supplement: Supplementary file 1 — Supporting Information [file ANIE-61-0-s001.pdf]

Angewandte Chemie
Eine Zeitschrift der Gesellschaft Deutscher Chemiker

Supporting Information

High-Field NMR, Reactivity, and DFT Modeling Reveal the γ-Al_2O_3 Surface Hydroxyl Network

N. Merle, T. Tabassum, S. L. Scott, A. Motta, K. Szeto, M. Taoufik, R. M. Gauvin*, L. Delevoye**

Supporting Information

Table of contents

General conditions S2

Figure S1. XRD diagrams of the γ-Al_2O_3 used in the present study S4

Figure S2. ^1H-^1H DQ-SQ MAS NMR spectra of **$Al_2O_{3\text{-}700}$-CO_2** S5

Figure S3. ^1H-^1H DQ-SQ MAS NMR spectra of **$Al_2O_{3\text{-}300}$**, **$Al_2O_{3\text{-}500}$** and **$Al_2O_{3\text{-}700}$** S6

Figure S4. Best fit simulations of the ^1H MAS NMR spectrum of **$Al_2O_{3\text{-}300}$**, **$Al_2O_{3\text{-}500}$** and **$Al_2O_{3\text{-}700}$** S7

Figure S5. Terminal hydroxyl region of ^1H-^1H DQ-SQ MAS NMR spectra of **$Al_2O_{3\text{-}300}$**, **$Al_2O_{3\text{-}500}$** and **$Al_2O_{3\text{-}700}$** S8

Figure S6. ^1H-^{27}Al D-HMQC MAS NMR spectra of **$Al_2O_{3\text{-}300}$**, **$Al_2O_{3\text{-}500}$** and **$Al_2O_{3\text{-}700}$** S8

Figure S7. ν(OH) region of the IR spectrum of **$Al_2O_{3\text{-}300}$**, **$Al_2O_{3\text{-}500}$** and **$Al_2O_{3\text{-}700}$** S9

Table S1. Evolution of ^1H NMR chemical shift for the different sites of **$Al_2O_{3\text{-}300}$**, **$Al_2O_{3\text{-}500}$** and **$Al_2O_{3\text{-}700}$** S9

Table S2. Best fit simulations for the ^1H NMR spectrum of **$Al_2O_{3\text{-}300}$**, **$Al_2O_{3\text{-}500}$** and **$Al_2O_{3\text{-}700}$** S10

Figure S8. Alkane metathesis catalyzed by **WH_{500}.** S11

Computational Details S11

Figure S9. Optimized structure of γ-Al_2O_3 and aluminum and oxygen sites exposed on the optimized alumina (*110*) surface S12

Figure S10. Combination of configurations **1** and **4** S13

General conditions

The γ-alumina (E7102 from Rhône-Poulenc) was first calcined at 500 °C under a N_2/O_2 flow overnight, then hydrated and heated at 100 °C. In a further step, the alumina was treated at the desired temperature (300, 500 or 700 °C) under high vacuum (10^{-5} Torr) for 12 hours. After this treatment, it was stored and handled in an argon-filled glove box. The specific surface area measured by BET (N_2) was 200 $m^2.g^{-1}$. The XRD spectrum (Figure S1) confirms the structural assignment as γ-Al_2O_3, in comparison with the diffraction pattern, and matches that of the reference 00-029-0063 (γ-Al_2O_3) from ICDD (International Center for Diffraction Data). Preparation of tungsten hydrides on γ-Al_2O_3 treated at various temperatures was performed by grafting of [W(≡C*t*Bu)(CH$_2$*t*Bu)$_3$] and subsequent hydrogenolysis to published procedures.[1] The W loading for **WH_{300}**, **WH_{500}** and **WH_{700}** is 4.3, 4.7 and 3.3 w%, respectively.

$Al_2O_{3\text{-}700}$-CO_2 : CO_2 adsorption on **$Al_2O_{3\text{-}700}$** was carried out at room temperature in a 500 ml Schlenk-type glass reactor. CO_2 (250 torr) was introduced in the reactor containing about 100 mg of **$Al_2O_{3\text{-}700}$** through a high vacuum line. Excess of CO_2 was removed under high vacuum at room temperature after 100 minutes of reaction. All the samples were stored under argon in a glovebox at ambient temperature.

$Al_2O_{3\text{-}700}$-Cl: chlorination of **$Al_2O_{3\text{-}700}$** was performed following a modified method described by Kytökivi.[2] A 1 g sample of **$Al_2O_{3\text{-}700}$** previously calcined was treated at 700 °C under high vacuum (10^{-5} Torr) for 12 h. Then a 1 L flask loaded with 600 Torr of gaseous HCl was connected to the vacuum line via a tee shape connector. Under static vacuum, HCl was allowed to react with alumina heated at 700 °C for 5 minutes. HCl was then condensed into the 1 L flask with liquid nitrogen and the alumina was treated at 700 °C under high vacuum for an additional 30 minutes.

Propane metathesis. The catalytic reaction was performed in a Microactivity reactor from PID Eng&Tech. In the glove box, the catalyst was charged into a stainless steel ½' cylinder reactor which can be isolated from atmosphere. Propane was purified via cartridges filled with molecular sieves and copper deoxo catalyst. After connection to the gas lines and purging of tubing, a flow of propane controlled by a Brooks® mass flow-controller (2.6 $molC_3$ $molW^{-1}$ min^{-1}) was passed onto the catalyst bed which was heated at 150 °C and 1 bar. Hydrocarbon products were analyzed on line by a HP 8890 chromatograph fitted with an Al_2O_3/KCl capillary column (50 m × 0.32 mm) and FID detector. The reaction was performed at 150 °C and 1 bar, the stainless steel reactor was connected to a Varian CP-3800 GC equipped with KCl/Al_2O_3 column and FID for product determination.

IR spectroscopy. IR spectra were taken at room temperature in diffuse reflectance mode in a Nicolet 6700 FTIR spectrophotometer equipped with MCT detector and SMART accessory DRIFT module. An airtight cell with CaF_2 window was employed, allowing sample preparation in the glovebox. Each spectrum comprised 64 scans with 4 cm^{-1} resolution. The background spectrum was previously recorded under the same conditions by placing a suitable mirror (provided by ThermoScientific) on the sample holder in the glovebox.

Solid state NMR. For solid-state NMR analysis, the highly air-sensitive samples were packed into 3.2-mm zirconia rotors inside an Ar-filled glovebox and sealed with tightly fitting Kel-F caps. ^{1}H MAS NMR, ^{1}H−^{1}H DQ-SQ MAS NMR and ^{1}H−^{27}Al *D*-HMQC MAS NMR spectra were acquired on a Bruker Avance III 800 spectrometer (^{1}H, 800.13 MHz; ^{27}Al, 208.50 MHz). For all experiments, the spinning frequency was 20 kHz. For ^{1}H MAS NMR spectra, 16 scans were collected using a π/2 pulse excitation of 3.0 μs and a recycling delay of 320 s to ensure full magnetization recovery and precise quantification. *D*-HMQC experiments were set up with an ^{27}Al spin echo selective to the central transition, with pulses of 8.5 and 17 μs, and a ^{1}H π/2 pulse of 2.75 μs on either side of the ^{27}Al π pulse. A recycling delay of 2 s was used. The $SR4_1^2$ dipolar recoupling scheme[3] was applied for 500 μs. The effect of dipolar recoupling time was probed, showing that optimal results were obtained with values between 500 μs and 1 ms. We chose 500 μs as a compromise between signal intensity and better selectivity towards first coordination sphere contribution. Longer recoupling time led to increased bulk signal, which is detrimental to spectral interpretation. Two-dimensional (2D) ^{1}H−^{1}H double-quantum single-quantum magic-angle spinning (DQSQ MAS) experiments were performed at 20 kHz spinning speed using the $R12_2^5$ symmetry-based recoupling scheme,[4] applied for 190 μs at a radio frequency field strength of 40 kHz. A total of 16 or 32 transients were summed for each of the 100 t1 increments, with a recycle delay of up to 100 s (for **$Al_2O_{3\text{-}300}$**, **$Al_2O_{3\text{-}500}$** and **$Al_2O_{3\text{-}700}$**).

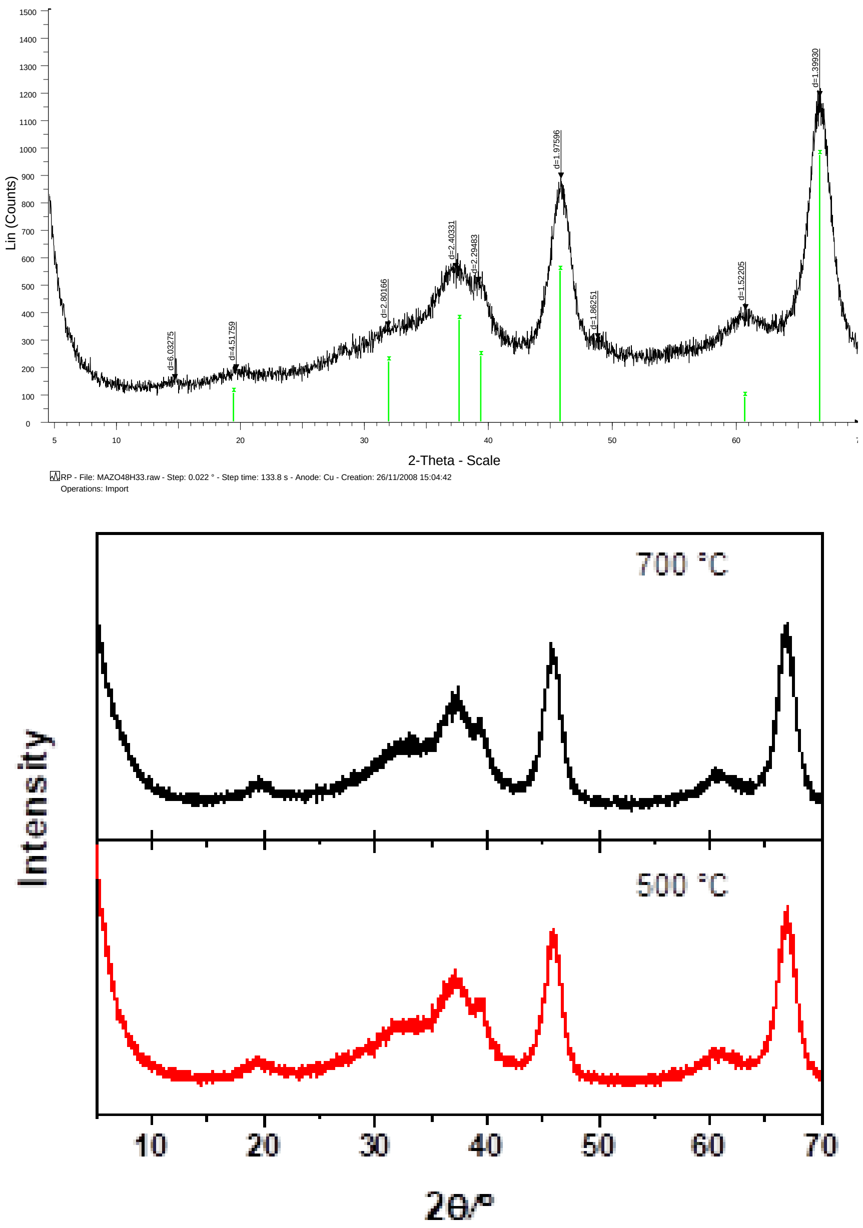

Figure S1. Top: XRD diagram of the γ-Al_2O_3 used in the present study and 00-029-0063 γ-Al_2O_3 (green lines); Bottom: XRD diagram of $Al_2O_{3\text{-}500}$ (red trace) and $Al_2O_{3\text{-}700}$ (black trace)

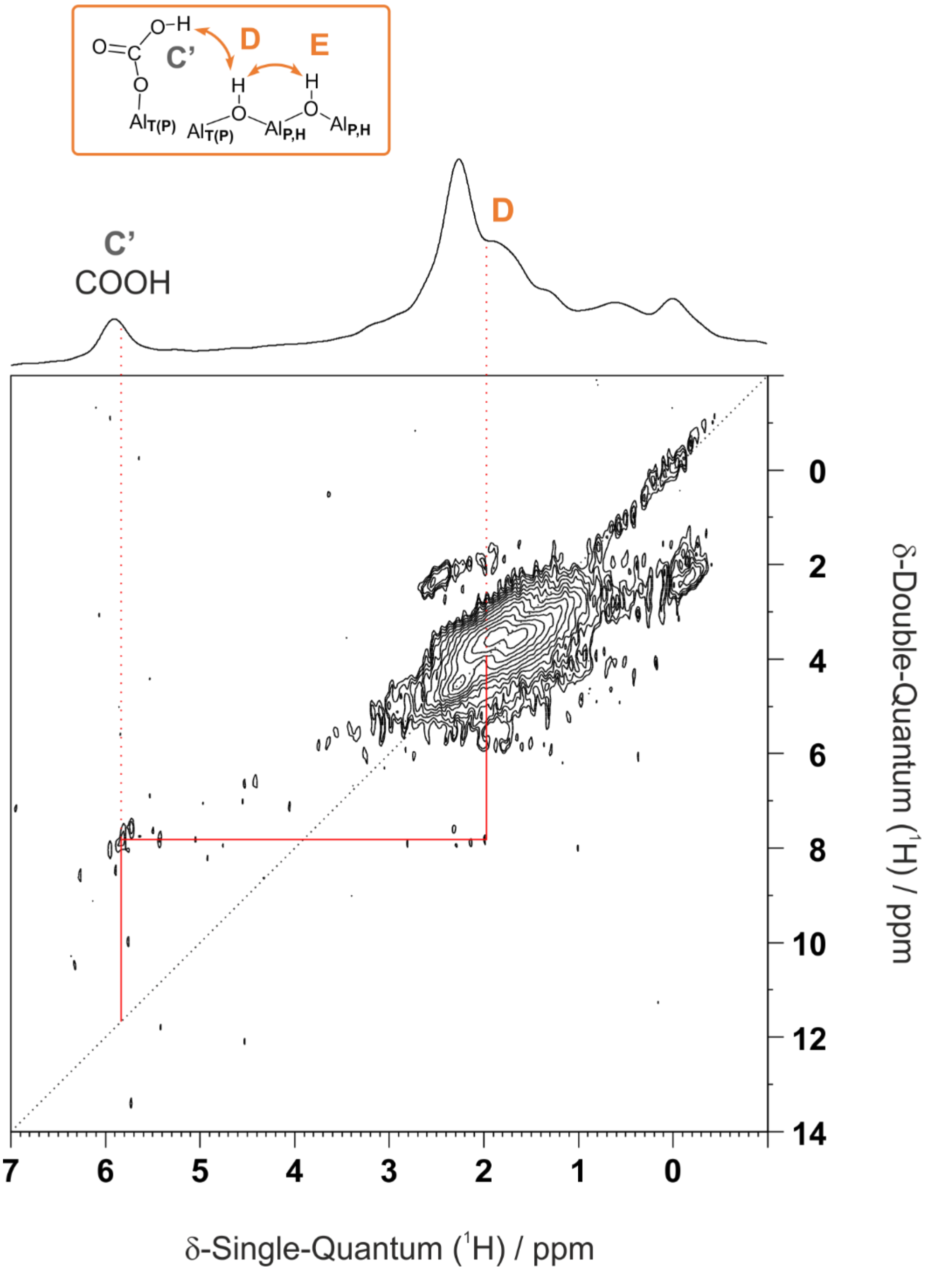

Figure S2. ^{1}H-^{1}H DQ-SQ MAS NMR spectra of **$Al_2O_{3\text{-}700}$-CO_2** illustrating the correlation between the carbonate OH **C'** and the site **D** (18.8 T, spinning speed 20 kHz).

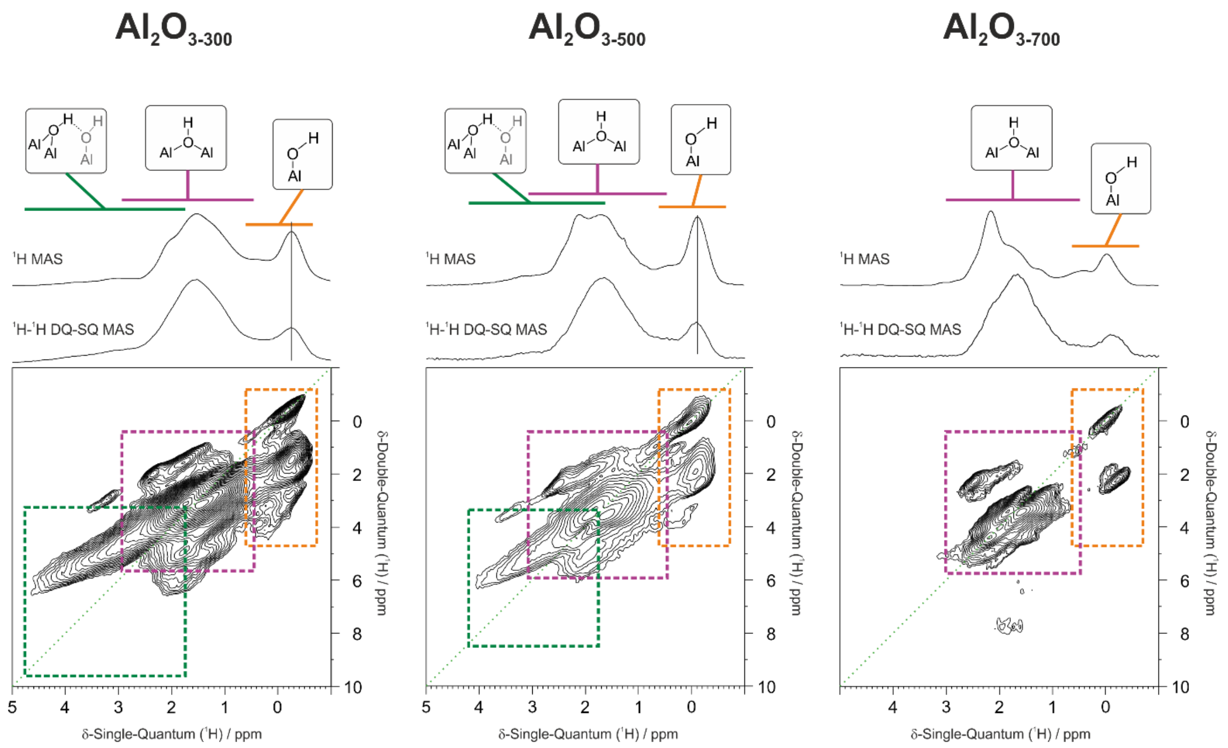

Figure S3. ^1H-^1H DQ-SQ MAS NMR spectra of **$Al_2O_{3\text{-}300}$** (left), **$Al_2O_{3\text{-}500}$** (center) and **$Al_2O_{3\text{-}700}$** (right), along with sums of rows (bottom 1D spectrum) and ^1H MAS NMR (top 1D spectrum) (18.8 T, spinning speed 20 kHz).

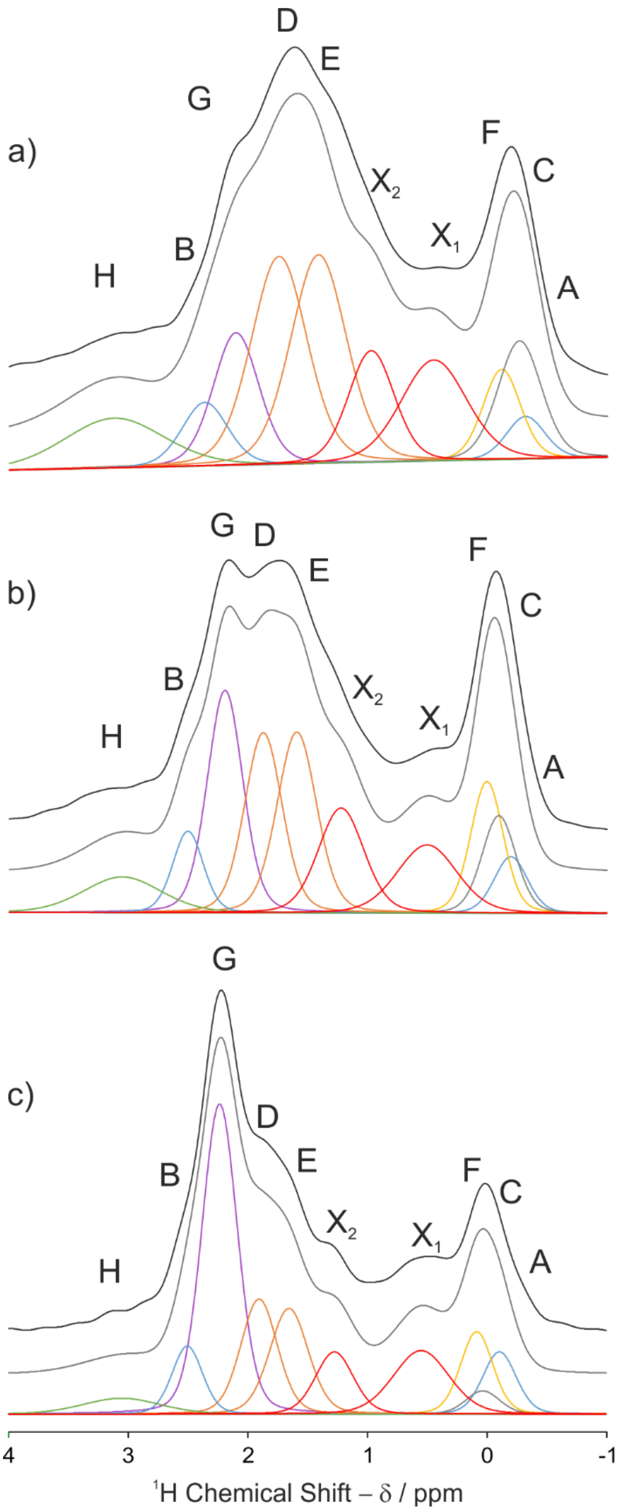

Figure S4. Best fit simulations of the ^{1}H MAS NMR spectrum of **$Al_2O_{3\text{-}300}$** (a), **$Al_2O_{3\text{-}500}$** (b) and **$Al_2O_{3\text{-}700}$** (c) (18.8 T, spinning speed 20 kHz).

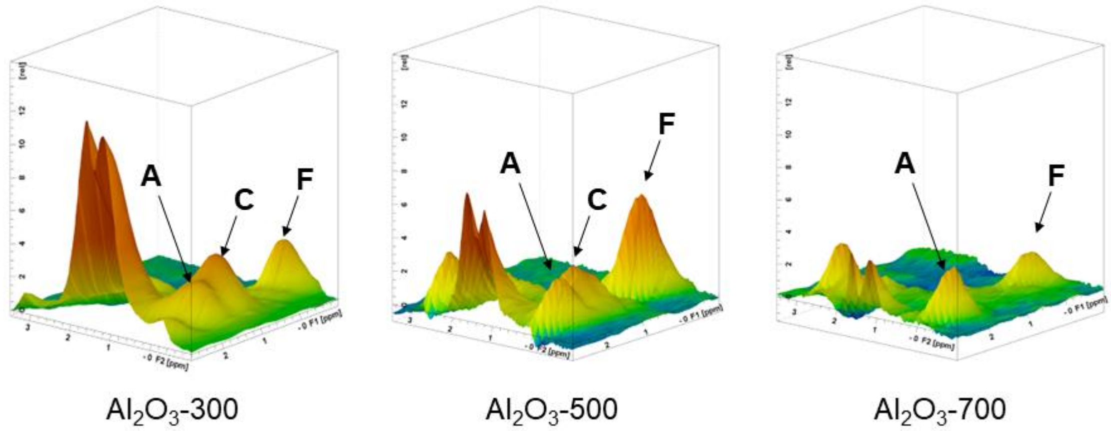

Figure S5. Terminal hydroxyl region of the ^1H-^1H DQ-SQ MAS NMR spectra of **$Al_2O_{3\text{-}300}$** (left), **$Al_2O_{3\text{-}500}$** (center) and **$Al_2O_{3\text{-}700}$** (right) (18.8 T, spinning speed 20 kHz).

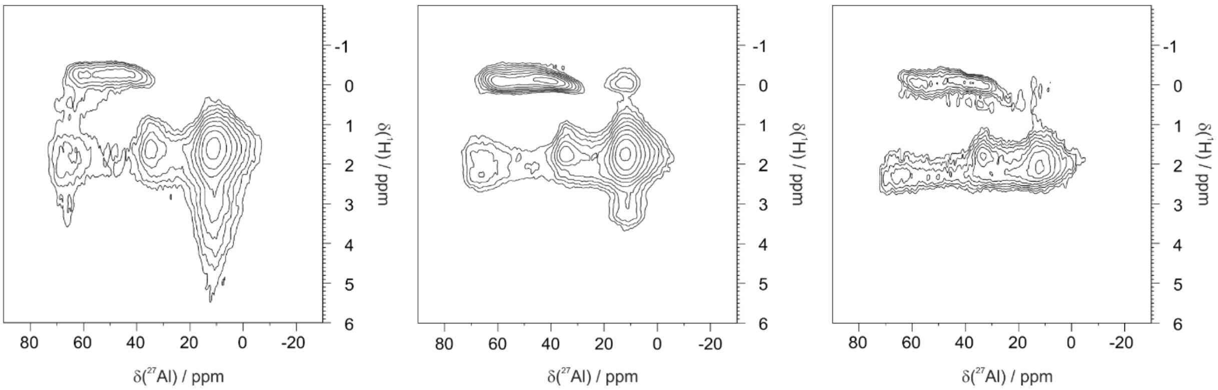

Figure S6. ^1H-^{27}Al D-HMQC MAS NMR spectra of **$Al_2O_{3\text{-}300}$** (left), **$Al_2O_{3\text{-}500}$** (center) and **$Al_2O_{3\text{-}700}$** (right) (18.8 T, spinning speed 20 kHz).

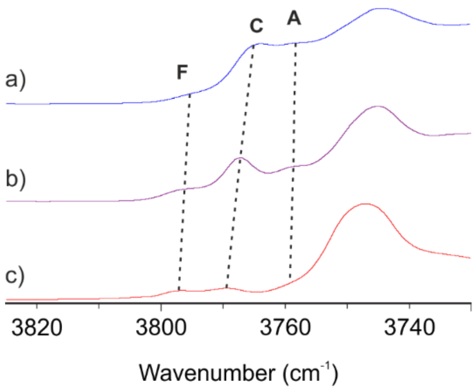

	$\nu_{(OH)}$, cm^{-1} F	C	A
**$Al_2O_{3\text{-}300}$**	3790	3769	3754
**$Al_2O_{3\text{-}500}$**	3794	3774	3759
**$Al_2O_{3\text{-}700}$**	3795	3778	3760

Figure S7. Evolution of the hydroxyl elongation region of the infrared spectrum of **$Al_2O_{3\text{-}300}$** (a), **$Al_2O_{3\text{-}500}$** (b) and **$Al_2O_{3\text{-}700}$** (c), along with the corresponding assignments for sites **A**, **C** and **F**.

Table S1. Evolution of ^{1}H NMR chemical shift for the different sites of **$Al_2O_{3\text{-}300}$**, **$Al_2O_{3\text{-}500}$** and **$Al_2O_{3\text{-}700}$** (from Figure S4).

	**$Al_2O_{3\text{-}300}$**	**$Al_2O_{3\text{-}500}$**	**$Al_2O_{3\text{-}700}$**
A	-0.32	-0.20	-0.11
B	2.36	2.50	2.50
C	-0.27	-0.10	0.03
D	1.74	1.87	1.90
E	1.41	1.59	1.65
F	-0.12	0.00	0.08
G	2.10	2.19	2.23
H	3.13	3.05	3.05
X1	0.45	0.50	0.54
X2	0.97	1.22	1.27

Table S2. Results of best fit simulations for the 1H NMR spectrum of **Al_2O_{3-300}**, **Al_2O_{3-500}** and **Al_2O_{3-700}** in % (from Figure S4).

	**Al_2O_{3-300}**	**Al_2O_{3-500}**	**Al_2O_{3-700}**
A	2.8	4.2	6.1
B	4.9	5.5	6.1
C	8.9	7.3	2.3
D	19.7	14.9	12.4
E	19.8	15.8	12.0
F	5.7	9.4	7.9
G	10.3	17.5	32.4
H	8.0	6.0	3.5
X1	11.2	8.9	10.4
X2	8.7	10.5	6.9
**Sum**	100	100	100

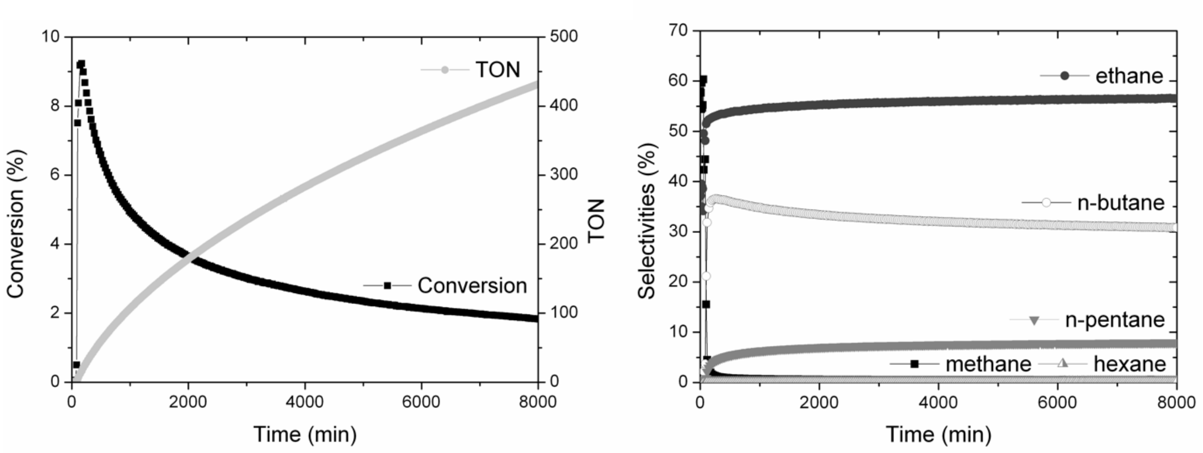

Figure S8. Alkane metathesis catalyzed by **WH_{500}**: Evolution of the turnover number and the product molar selectivity for the test performed at 150 °C, 6 bar, 4 mL min^{-1} propane.

Computational details

DFT-based simulations were performed with the CP2K/Quickstep package, using a hybrid Gaussian and plane wave method.[5] A double quality DZVP Gaussian basis set was employed for all atoms. The Goedecker-Teter-Hutter[6] pseudopotentials together with a 400 Ry plane wave cutoff were used to expand the densities obtained with the Perdew-Burke-Ernzerhof (PBE) [7] exchange-correlation density functional. Molecular graphics were produced by the CHEMCRAFT graphical package. [8] NMR chemical shifts and the electric field gradient was evaluated by the GIPAW module within the quantum expresso package. [9,10] The nuclear electric quadrupole moment for oxygen and hydrogen atoms are taken from Stone. [11] For ^{27}Al we use a Q value of -8.0178 x 10^{-30} m^2. [12]

Surfaces model. The γ-alumina bulk model used in this study is taken from theoretical investigations of Digne et al. [13] The structure (Figure S9, left) contains a fcc sublattice of oxide ions that generates octahedral and tetrahedral interstices which accommodate aluminum ions.

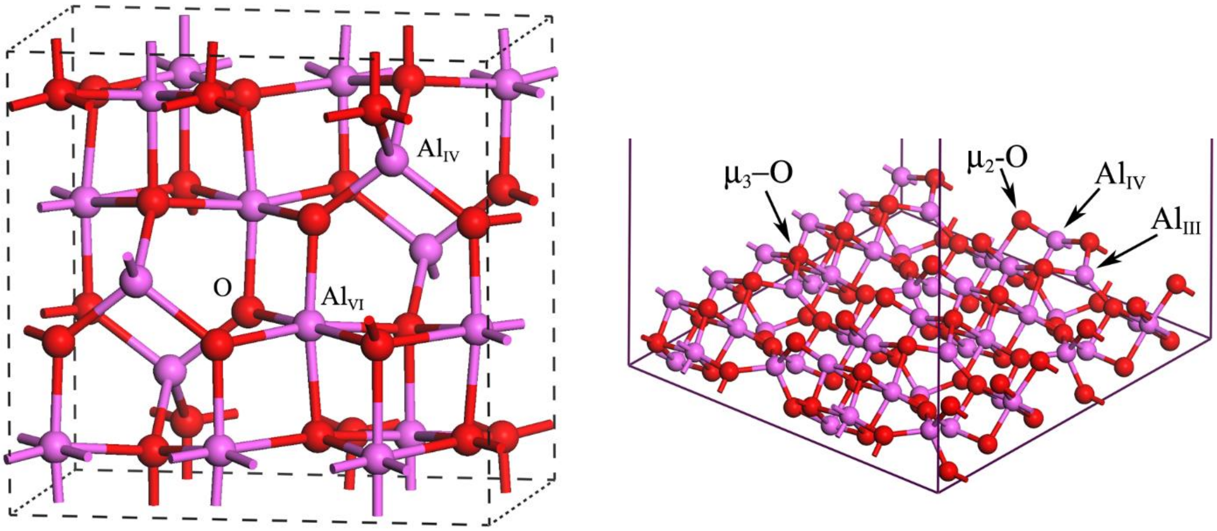

Figure S9. Left: Optimized structure of γ-alumina. Right: Aluminium and oxide sites exposed on the optimized alumina (*110*) surface.

Recent experimental data[14] for γ-alumina indicate that, in a spinel-type indexing, the (*110*) surface predominates even though it undergoes a significant reconstruction, forming nanoscale (111) Al_2O_3 facets. Thus, the present analysis focuses on the (*110*) surface since it represents the principal exposed crystallographic surface. The surface consists of a (2×2) surface unit cell (16.778 Å x 16.187 Å) having a slab of seven reticular plane thickness (216 O atoms and 144 Al atoms) and repeated periodically with a 20 Å vacuum region between the slabs. The deepest layer was frozen to simulate the bulk description. The optimized alumina (*110*) surface was found to exhibit significant rearrangement of Al and O ions relative to the bulk: namely bulk octahedral Al centers become pseudotetrahedral (Al_{IV} in Figure S9, right) while bulk tetrahedral Al centers become pseudo-trigonally planar on the surface (Al_{III} in Figure S9, right). Therefore, the surface O ions are found to have either μ_3-O and μ_2-O geometries. The μ_3-O species are bound to Al_{III} and Al_{IV} surface ions and to the octahedral Al_{VI} bulk ion, while the μ_2-O species are bound to the Al_{IV} and to the octahedral Al_{VI} bulk ions.

From experimental data the hydroxyl density of alumina annealed at 700°C is about 1.1 OH/nm^2. Then, simulations involve the absorption of either one or two water molecules on the modelled surface. In fact, with one adsorbed water molecule we obtain a 0.7 OH/nm^2 value of the hydroxyl density and with two water molecules we obtain a 1.5 OH/nm^2 value of the hydroxyl density.

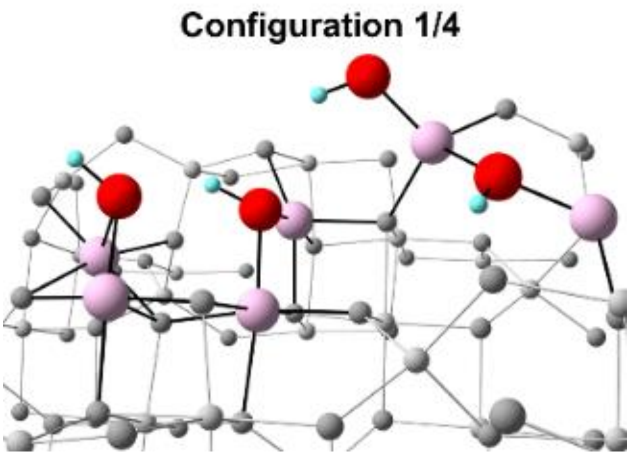

Figure S10. Combination of configurations **1** and **4** accounting for adsorption of two proximal water molecules.

---

[1] E. Le Roux, M. Taoufik, C. Coperet, A. de Mallmann, J. Thivolle-Cazat, J.-M. Basset, B. M. Maunders, G. J. Sunley, *Angew. Chem. Int. Ed.* **2005**, *44*, 6755-6758

[2] A. Kytökivi, M. Lindblad, A. Root, *J. Chem. Soc., Faraday Trans.* **1995**, *91*, 941–948.

[3] A. Brinkmann, A. P. M. Kentgens, Proton-Selective 17O-1H Distance Measurements in Fast Magic-Angle-Spinning Solid-State NMR Spectroscopy for the Determination of Hydrogen Bond Lengths. *J. Am. Chem. Soc*. **2006**, *128*, 14758.

[4] M. Carravetta, M. Eden, X. Zhao, A. Brinkmann, M. H. Levitt, Symmetry Principles for the Design of Radiofrequency Pulse Sequences in the Nuclear Magnetic Resonance of Rotating Solids. *Chem. Phys. Lett*. **2000**, *321*, 205.

[5] The CP2K developers group. http://www.cp2k.org

[6] S. Goedecker, M. Teter, J. Hutter, *Phys Rev B* **1996**, *54*, 1703-1710.

[7] J. P. Perdew, K. Burke, M. Ernzerhof, *Phys Rev Lett* **1996**, *77*, 3865-3868.

[8] The chemcraft developers group. https://www.chemcraftprog.com

[9] Giannozzi, P.; Baroni, S.; Bonini, N.; Calandra, M.; Car, R.; Cavazzoni, C.; Ceresoli, D.; Chiarotti, G. L.; Cococcioni, M.; Dabo, I.; et al. QUANTUM ESPRESSO: A Modular and Open-Source Software Project for Quantum Simulations of Materials. *J. Phys. Condens. Matter* **2009**, *21* (39), 395502.

[10] Giannozzi, P.; Andreussi, O.; Brumme, T.; Bunau, O.; Buongiorno Nardelli, M.; Calandra, M.; Car, R.; Cavazzoni, C.; Ceresoli, D.; Cococcioni, M.; et al. Advanced Capabilities for Materials Modelling with Quantum ESPRESSO. *J. Phys. Condens. Matter* **2017**, *29* (46), 465901.

[11] Stone J.N. *Atomic data and nuclear data tables*, **2016**, *111-112*, 1-28.

[12] Taoufik, M.; Szeto, K. C.; Merle, N.; Rosal, I. Del; Maron, L.; Trébosc, J.; Tricot, G.; Gauvin, R. M.; Delevoye, L. Heteronuclear NMR Spectroscopy as a Surface-Selective Technique: A Unique Look at the Hydroxyl Groups of γ-Alumina. *Chem. - A Eur. J.* **2014**, *20* (14), 4038–4046.

[13] Digne, M.; Sautet, P.; Raybaud, P.; Euzen, P. ; Toulhoat, H. *J. Catal.* **2004**, *226*, 54-68.

[14] Kovarik, L.; Genc, A.; Wang, C.; Qiu, A.; Peden, C. H. F.; Szanyi, J.; Kwak, J. H. *J. Phys. Chem. C* **2013**, *117*, 179–186.
